# Direct Visualization of Ultrastrong Coupling between Luttinger-Liquid Plasmons and Phonon Polaritons

**DOI:** 10.1021/acs.nanolett.1c04807

**Published:** 2022-03-22

**Authors:** Gergely Németh, Keigo Otsuka, Dániel Datz, Áron Pekker, Shigeo Maruyama, Ferenc Borondics, Katalin Kamarás

**Affiliations:** † Wigner Research Centre for Physics, Konkoly Thege Miklós út 29-33, 1121 Budapest, Hungary; ‡ Budapest University of Technology and Economics, Műegyetem rkp. 3, 1111 Budapest, Hungary; § Department of Mechanical Engineering, The University of Tokyo, Tokyo 113-8656, Japan; ∥ Eötvös Loránd University, Pázmány Péter sétány 1/A, 1117 Budapest, Hungary; ⊥ Synchrotron SOLEIL, L’Orme des Merisiers, 91192 Gif Sur Yvette CEDEX, France

**Keywords:** s-SNOM, near-field, infrared, plasmon, phonon, Luttinger-liquid, carbon nanotube, ultrastrong coupling

## Abstract

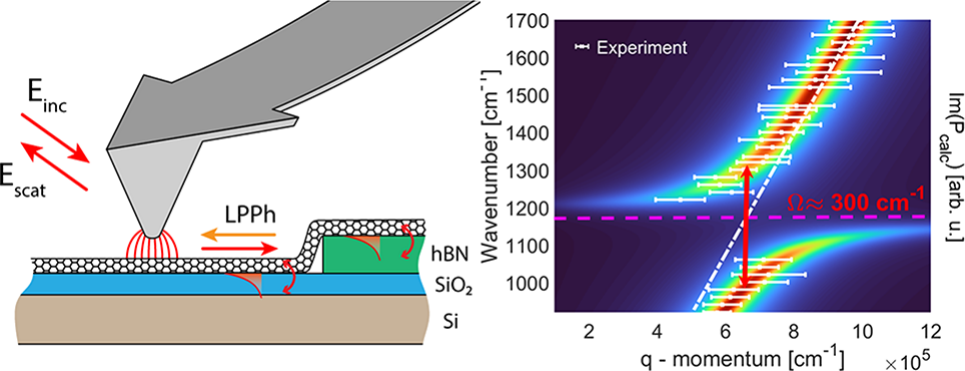

Ultrastrong coupling of light and matter creates new opportunities to modify chemical reactions or develop novel nanoscale devices. One-dimensional Luttinger-liquid plasmons in metallic carbon nanotubes are long-lived excitations with extreme electromagnetic field confinement. They are promising candidates to realize strong or even ultrastrong coupling at infrared frequencies. We applied near-field polariton interferometry to examine the interaction between propagating Luttinger-liquid plasmons in individual carbon nanotubes and surface phonon polaritons of silica and hexagonal boron nitride. We extracted the dispersion relation of the hybrid Luttinger-liquid plasmon–phonon polaritons (LPPhPs) and explained the observed phenomena by the coupled harmonic oscillator model. The dispersion shows pronounced mode splitting, and the obtained value for the normalized coupling strength shows we reached the ultrastrong coupling regime with both native silica and hBN phonons. Our findings predict future applications to exploit the extraordinary properties of carbon nanotube plasmons, ranging from nanoscale plasmonic circuits to ultrasensitive molecular sensing.

Surface plasmon polaritons (SPPs) are hybrid light–matter quasiparticles formed by the coupling of electromagnetic waves and collective charge oscillations.^1,2^ They enable subwavelength trapping of light that yields extreme concentration of the electromagnetic field, enabling nanoscale modification of light–matter interactions.^3^ Low-dimensional materials such as 2D van der Waals structures and one-dimensional nanotubes support various polaritonic excitations from the mid-infrared to the visible range.^4−10^ Low-dimensional plasmons have the advantage of high confinement ratio and easy tunability by the dielectric environment or carrier density.^11−16^ A particularly important phenomenon is strong coupling of quasiparticles which permits applications like induced transparency, polariton lasing, changing of the rate of chemical reactions, or enhanced sensitivity in infrared and Raman spectroscopy.^17−23^ Strong coupling of low-dimensional plasmons was demonstrated by showing hybridization of graphene plasmons with phonon polaritons of various substrates.^24−26^ Importantly, graphene plasmons were also exploited to enhance the sensitivity of infrared and Raman spectroscopy, but only localized modes were studied.^27−29^

Carbon nanotubes (CNTs) can provide an even higher degree of electromagnetic field confinement than graphene.^31^ As CNT electrons are constrained in one dimension, their interaction exerts a significant influence on the collective behavior of conduction electrons. The strong electron–electron correlation results in the Luttinger-liquid state of Dirac electrons.^32^ Multiple experiments proved the existence of the Luttinger-liquid state in carbon nanotubes from photoemission,^33^ NMR,^34^ and ESR^35^ to electrical transport.^36^ Recently, Luttinger plasmons were also visualized in real space by near-field microscopy.^37^ Because of the Luttinger-liquid state, the scattering channels of CNT plasmons are limited, and thus, they are coherent excitations with high quality factor.^38−40^ The linear dispersion of Luttinger-liquid plasmons, which depends on the Fermi velocity *v*_f_, places their excitation frequency into the mid-infrared.^41^ These exceptional properties of CNT Luttinger-liquid plasmons make them ideal candidates to observe strong coupling with other quasiparticles.

Plasmon–phonon resonances of a CNT thin film and SiO_2_ substrate were demonstrated by Falk et al.^42^ using far-field spectroscopy with specially engineered samples. However, the interaction of propagating Luttinger-liquid plasmons and surface phonon polaritons at the individual nanotube level has not been studied so far.

Here, we demonstrate real-space imaging of ultrastrong coupling between Luttinger-liquid plasmons in individual carbon nanotubes and a thin native silicon oxide (silica) layer using near-field microscopy. This method provides spatial information at the scale of the plasmon wavelength and reveals the impact of the local environment on the plasmon propagation as well. Our results show that plasmons in individual nanotubes are significantly more confined than in nanotube ensembles,^42^ and we also analyze the strength of coupling more rigorously. With the help of near-field polariton interferometry, we extract the dispersion relation of the plasmon–phonon hybrid system which shows anticrossing and mode splitting. To evaluate the coupling, we used the classical coupled harmonic oscillator model to fit both the plasmon spectrum and the dispersion relation. The interaction can be modified by increasing the distance of the nanotube from the silica layer by inserting a hexagonal boron nitride (hBN) flake between the nanotube and the substrate.

Figure 1a illustrates the schematics of our experiments. To study CNT plasmons in individual nanotubes, we applied scattering-type near-field infrared microscopy (s-SNOM). In our instrument (NeaSNOM, Neaspec GmbH) a metal-coated atomic force microscope (AFM) tip (Arrow-NCPt, Nanoworld) is illuminated by a focused laser beam of a tunable infrared quantum cascade laser (MIRCat QT, Daylight Solutions). The illuminated tip acts as a nanoscale light source used to locally probe the optical properties of the sample. The AFM is working in tapping mode, and the scattered light arising from the tip–sample near-field interaction is detected by the combination of higher harmonic demodulation and pseudoheterodyne interferometry (PsHet). In a typical measurement, the AFM topography and near-field optical images are collected simultaneously. The PsHet detection permits acquisition of both the amplitude (*s*_*n*_) and the phase (φ_*n*_) of the scattered light, where the subscript denotes the demodulation order.^43^

**Figure 1:**
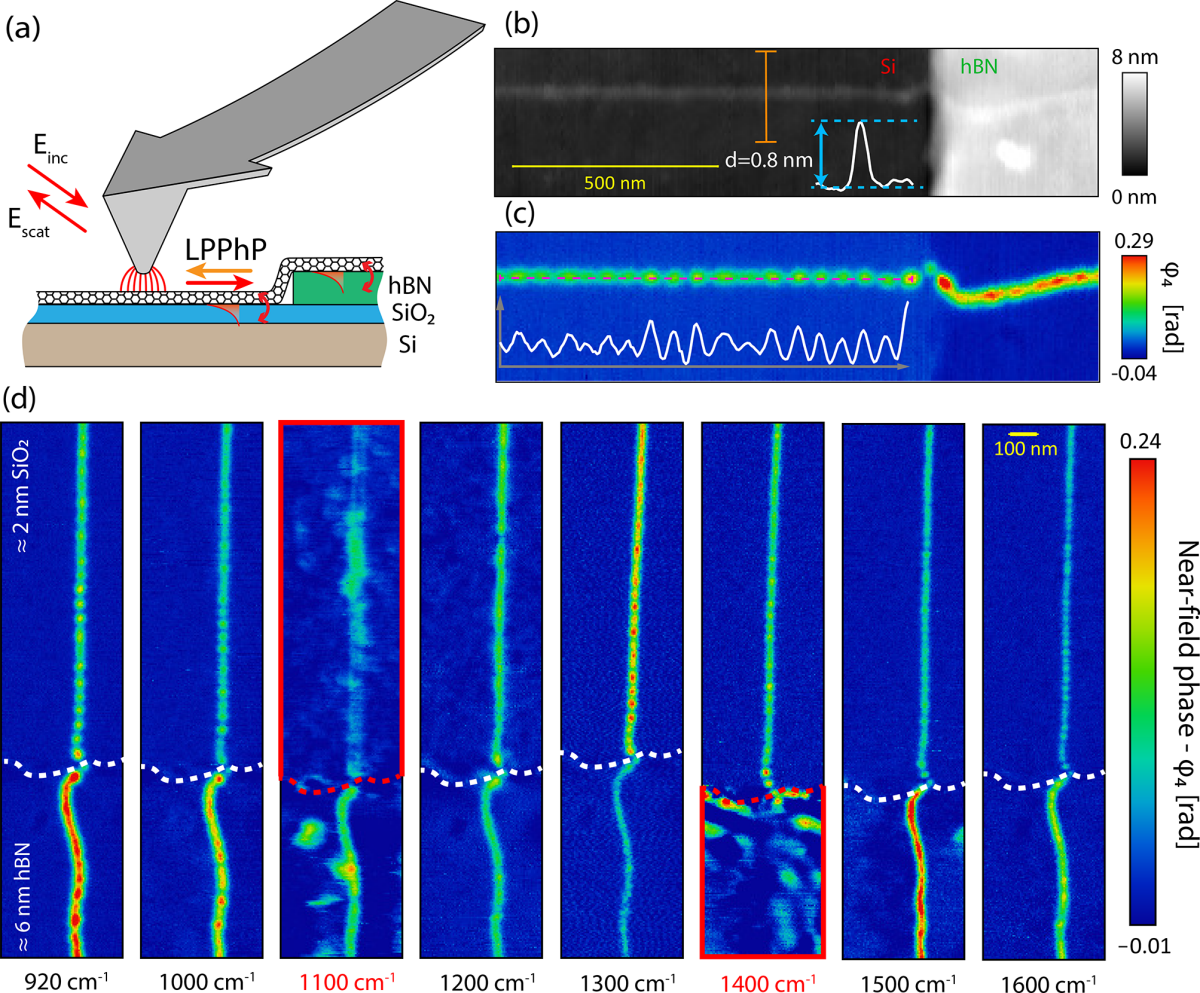
(a) Schematic illustration of nanotube polariton imaging with s-SNOM. (b) AFM topography of an individual nanotube partially on silicon/native silica and partially on a 6 nm thick hBN flake. Also shown is a line profile across the nanotube along the orange line, yielding a nanotube diameter of 0.8 nm. (c) Corresponding near-field phase image (φ_4_) taken at 920 cm^–1^. Phase values were normalized to that of silicon. The inset shows the profile extracted along the dashed purple line. We note that the first less intense spot at the material boundary was excluded from the profile. Its lower intensity is caused by the phase shift upon reflection, discussed in the Supporting Information.^30^ (d) Near-field phase images at several different laser frequencies. In the map taken at 1100 cm^–1^, the phase contrast of the nanotube on silicon is near zero and the plasmon fringes are missing. On the other hand, the phase contrast on hBN is still apparent but vanishes at 1400 cm^–1^. These two frequencies correspond to the Reststrahlen band of silica and hBN, respectively, and highlight that plasmons are coupled to the phonons of each substrate yielding a significant dip in the near-field phase spectrum.

The optical near fields generated at the apex of the tip have sufficiently high momentum to launch propagating plasmons. The tip-launched plasmons propagate along the nanotube and can reflect on different defects or perturbations (edges, geometric or dielectric obstacles). The reflected plasmons then propagate back to the tip and interfere with the forward propagating ones. This results in plasmon interference fringes along the direction of propagation. By raster scanning of the sample, plasmons can be visualized through their interference fringes. This so-called polariton interferometry was previously used to examine various polaritonic effects in low-dimensional materials.^41,44,45^

Long, parallel, and straight individual single-walled carbon nanotubes were grown by chemical vapor deposition (CVD) on quartz and transferred subsequently via PMMA support film onto an undoped silicon substrate with thin hBN flakes previously exfoliated onto the surface. PMMA was washed away by acetone, and the sample was annealed in Ar atmosphere at 350 °C (details in Supporting Information section 2).

In this study, we concentrate on metallic carbon nanotubes as they support strong tip-launched Luttinger-liquid plasmons at ambient conditions, in contrast to semiconducting ones that require additional doping.^46^ The metallic nanotubes can be easily identified based on their very pronounced near-field phase contrast compared to semiconducting ones..^47−49^ Additionally, phase contrast is much more resilient to the topographic artifacts whereas the amplitude is very sensitive to the tip–sample distance variations (Supporting Information section 1). On the basis of these findings, we present only near-field phase maps in this article.

Figure 1b shows a representative nanotube that is located partially on the Si/SiO_2_ surface (left side) and partially on an hBN flake (right side). As the profile shows, the nanotube itself is 0.8 nm in diameter. The hBN flake height is 6 nm which separates the nanotube from the Si/SiO_2_ surface. The fourth harmonic demodulated phase (φ_4_) was acquired simultaneously and is plotted in Figure 1c. The plasmons launched by the tip reflect back from the material boundary: it serves as a geometric obstacle as the nanotube is distorted where it reaches the hBN flake. The fringe pattern arising from the plasmon interference is clearly visible in both the Si/SiO_2_ and the hBN domain. Figure 1c also contains the near-field phase line profiles taken along the purple line. The plasmon wavelength λ_p_ is then simply calculated as twice the distance between adjacent peaks. For the illumination frequency of 920 cm^–1^ the plasmon wavelength on the Si/SiO_2_ substrate becomes λ_p_ = 109.2 ± 3.8 nm, in good agreement with previously reported values.^41^ The small discrepancy between these and previous experiments originates from the exact plasmon wavelength being a function of the nanotube diameter and the dielectric environment.^41^

To spectrally study the plasmon excitation and to retrieve the plasmon dispersion, we tuned the laser frequency from 920 to 1700 cm^–1^ in 20 cm^–1^ steps. By reimaging the same area at each laser frequency, we determined the ω(*q*) nanotube plasmon dispersion, where *q* = 2π/λ_p_ is the plasmon wavevector corresponding to its momentum. Upon near-field excitation, the plasmon momentum has to match the in-plane wavevector of a tip-generated near-field component. In Figure 1 d we present several near-field phase maps that highlight the spectral characteristics of the plasmon excitations. In each map, phase values are normalized to that of the underlying substrate by calculating φ_4_ = φ_4,CNT_ – φ_4,subs_. This way we can remove phase differences between the substrates and we focus only on the nanotube signal.

Two important effects are apparent already from the near-field maps. First, the periodicity of the plasmon interference fringes changes with excitation frequency, unraveling the ω(*q*) relationship. Further, the amplitude of the oscillations also depends on the excitation energy. There are two very prominent maps (1100 and 1400 cm^–1^) where the phase contrast completely vanishes: these are the ranges of SiO_2_ and hBN phonons, respectively. The Reststrahlen band of a medium that supports lattice vibrations is located between the TO and LO phonon frequencies where the real part of the dielectric function is negative. For SiO_2_ the frequencies are ω_TO_ = 1071 cm^–1^ and ω_LO_ = 1184 cm^–1^,^50^ while for hBN ω_TO_ = 1378 cm^–1^ and ω_LO_ = 1610 cm^–1^.^51^ We attribute the lack of phase contrast, thus excited states, to the hybridization between surface phonon modes of the thin phononic layer material (specifically native silica and hBN flake) and carbon nanotube plasmons. First, we analyze more thoroughly the effect of native silica.

From all near-field images, we extracted a line profile of the plasmon fringes along the nanotube, similar to Figure 1c. Figure 2c depicts the amplitude of the Fourier transform of the plasmon fringes for all excitation frequencies assembled in a frequency–momentum map. Additionally, we manually determined the distances between adjacent maxima of the fringe profiles and superimposed the manually calculated plasmon wave vector values. The significant lack of modes is well aligned with the Reststrahlen band of silica (marked by the orange dashed lines in Figure 2) where thin-film phonon modes can be excited. In this range, from 1070 to 1200 cm^–1^, plasmon oscillations are suppressed and the corresponding components are missing from the Fourier spectrum. The manually measured plasmon wavevector values are also missing because there are no recognizable oscillations in the images.

**Figure 2:**
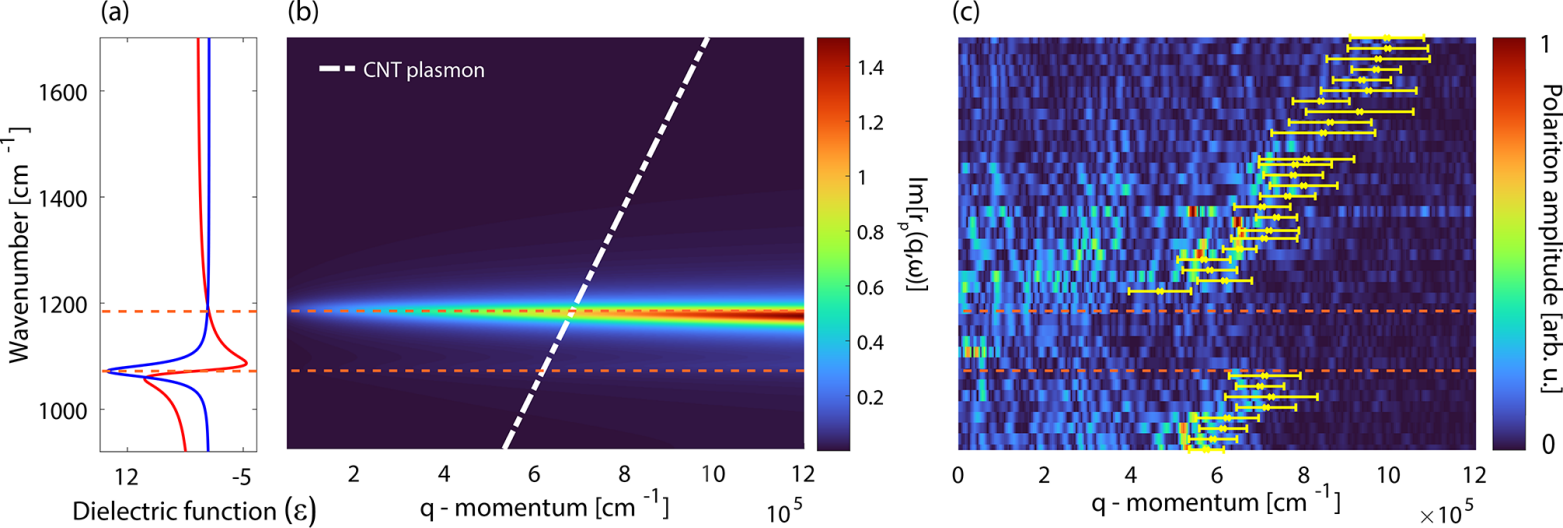
(a) Dielectric function of SiO_2_ with the orange dashed lines marking the Reststrahlen band. (b) Imaginary part of the Fresnel reflection coefficient of a 2 nm thick native silica layer on undoped silicon. It shows a pronounced excitation that corresponds to the air–silica interface phonon mode. (c) Color plot presenting the amplitude of the Fourier transform of plasmon interference fringes taken along the nanotube for each illumination frequency (dispersion map) together with the manually measured wavevector values with error bars (yellow plot).

We determined the native silica layer thickness to be 2.17 nm by ellipsometry. The thin silica film supports surface phonon–polariton modes both at the air–silica (close to ω_LO_) and silica–silicon interfaces (close to ω_TO_).^52,53^Figure 2b depicts the imaginary part of the Fresnel reflection coefficient of a SiO_2_/Si system for optical fields in the same momentum and frequency range as in the experiments. The air–silica interface phonon mode is clearly more prominent than the silica–silicon mode. This mode has a polarization mostly perpendicular to the surface; thus it matches the polarization of the nanotube induced by the AFM tip. As the nanotubes are located at this interface, we consider it to play the dominant role in coupling.

The dispersion relation of Luttinger plasmons in metallic carbon nanotubes is linear: ω_p_ = *v*_p_·*q*, where the plasmon velocity *v*_p_ depends on the Luttinger-liquid interaction parameter *G* via *v*_p_ = *v*_f_/*G* (white dashed line in Figure 2 b).^41^*v*_f_ ≈ 0.8 × 10^6^ m/s represents the Fermi velocity in metallic carbon nanotubes. Owing to the wide distribution of optical near fields at the apex of the AFM tip, there is available momentum at every photon frequency to launch plasmons which can electromagnetically couple to surface modes of the oxide forming new hybrid Luttinger-liquid plasmon–phonon polaritons (LPPhPs).

We analyze and confirm the hybridization by the classical coupled harmonic oscillator model and take into account the nanotube plasmons and the air–silica interface phonon mode as two coupled harmonic oscillators. This system has eigenmodes significantly different from those of the original oscillators and can be calculated according to^54^
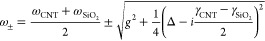
1Here ω_CNT_ and  are the resonance frequencies of the uncoupled oscillators and γ_CNT_ and  are the damping parameters. Δ = ω_CNT_ –  describes the detuning between the nanotube plasmon resonance and the silica phonon mode. During the experiments, we tune the nanotube plasmons according to their linear dispersion. The slope of its dispersion line is given by the plasmon velocity, which we found to be *v*_p_ = 3.32 × 10^6^ m/s. The damping was determined in two different ways. First, we fitted a Lorentzian curve to a vertical line cut from the measured dispersion map in Figure 2c at *q* = 7 × 10^5^ cm^–1^ which is away from zero detuning but still provides sufficient signal-to-noise ratio for the fit. Next, we fitted hybrid polariton peaks at zero detuning by the sum of two Lorentzian functions which provided
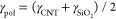
We get the same value, γ_CNT_ = 150 cm^–1^, with both approaches (details in Supporting Information section 5). The oscillator parameters for the silica slab mode were taken from a vertical line from Im(*r*) at *q* = 6.65 × 10^5^ cm^–1^. The frequency of the peak is  = 1176.1 cm^–1^ and  = 30 cm^–1^. With these oscillator parameters the coupling strength *g* is the only free parameter. We found that *g* = 150 cm^–1^ provides excellent agreement between experiments and the calculated dispersion. The result is shown in Figure 3a by the red dashed lines. As the coupling strength (*g*) exceeds the damping of both oscillators, the exchange of energy between them is faster than its leakage.

**Figure 3:**
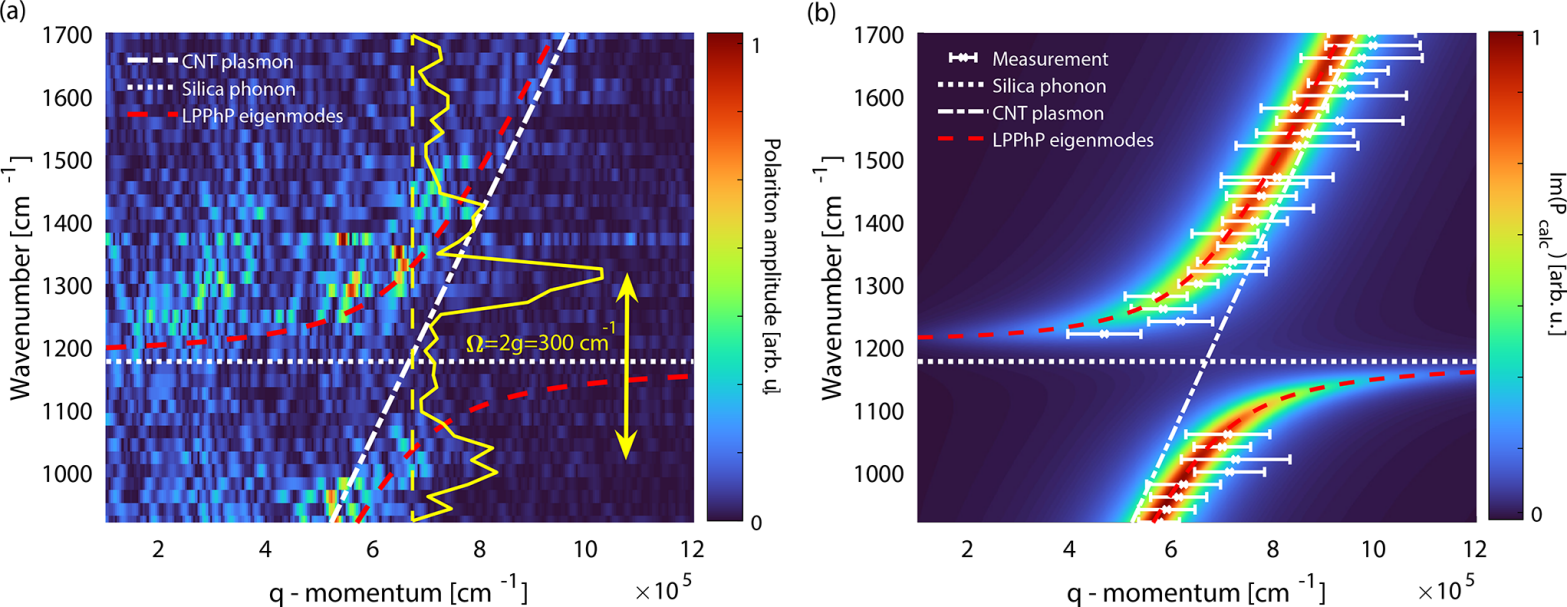
(a) Each row in the color plot presents the Fourier transform of the plasmon interference fringes taken at the corresponding illumination frequency (dispersion map). The white dashed lines show the bare plasmon dispersion and the silica surface phonon mode. The red dashed line plots the eigenmode frequencies of the hybridized Luttinger-liquid plasmon–phonon polariton (LPPhP) states considering coupling strength *g* = 150 cm^–1^. The lines properly match the dispersion map. At zero detuning, the mode splitting corresponds to Ω = 2*g*. We also plot the polariton amplitude taken as a vertical line cut at zero detuning (yellow plot). (b) Dispersion map of LPPhP hybrid states calculated by using the harmonic oscillator model. The manually determined plasmon wavevector values (with white error bars) are superimposed onto the map showing excellent agreement. Red dashed dispersion lines were calculated by eq 3.

For further analysis, we reproduced the full dispersion map with the coupled harmonic oscillator model. We assume that the tip-induced near field provides the driving force (*F* = *eE*_loc_), and only the nanotube plasmon is excited. The plasmon then exchanges energy with the phonon mode of the silica slab through electromagnetic coupling. The equation of motion of the system is described by a linear differential equation system^55−58^ (Supporting Information section 6). The steady-state solution for the displacement of the driven oscillator gives the polarization induced by the plasmon excitation *P* = *ex* and can be written as
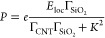
2where Γ_*j*_ = ω_*j*_^2^ – *iγ*_*j*_ω – ω^2^ is the frequency-dependent response of each oscillator and *K* = 2*giω* is the coupling term. The amplitude of each polariton fringe is proportional to the local absorption; thus we visualize the dispersion by plotting Im(*P*(ω, *q*)). Figure 3b shows the calculated dispersion map with the manually measured plasmon wavevector values. All calculations were done with *g* = 150 cm^–1^.

The coupling regime can be determined by comparing the coupling strength to the damping of the oscillators. The strong-coupling criterion is defined by
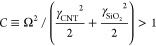
where Ω = 2*g* (mode splitting).^17^ By using the presented oscillator parameters, we obtain *C* = 7.7 that fulfills this requirement. Furthermore, applying another measure, we calculate the normalized coupling strength η = *g*/ω_*g*_ where ω_*g*_ = 1176.1 cm^–1^ is the mid-gap frequency. The result of η = 0.13 > 0.1 shows that the hybridization between propagating nanotube Luttinger-liquid plasmons and silica phonons reaches the ultrastrong coupling regime;^59^ thus, the damping can be neglected when calculating the eigenfrequencies. While eq 1 is an approximation for the eigenfrequencies, in this case we can formulate the exact solution as^17,58^
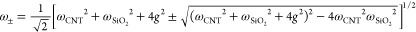
3Dispersion curves calculated this way are displayed in Figure 3b as red dashed lines. They align well with the maxima of the theoretical dispersion map. We note that frequencies given by eq 3 match those suggested by the quantum-mechanical Hopfield model as shown previously.^55,57,58,60^

To achieve ultrastrong coupling at mid-infrared frequencies is challenging because of weak oscillator strengths.^60^ Generally,  where *N* is the number of dipoles coupled to the electric field mode and *V* is the mode volume of the electric field. We attribute the high coupling strength to the extreme concentration of electromagnetic field around the nanotube. Previous studies estimated the electric field of the nanotube plasmon via finite element simulations and showed that the electric field distribution is concentrated to the close proximity of the nanotube surface.^15,61^ Its electric field decays on the scale of the nanotube diameter; thus in our case, the plasmon field is strongly confined into the 2 nm thick silica layer. The obtained value for η marks the exceptional properties of nanotube plasmons allowing ultrastrong coupling in the mid-infrared.

It is important to note that the polariton interference fringes cannot be observed properly in every measurement. For example, the phase contrast of nanotubes on the hBN flake does not present recognizable oscillations along the nanotubes for all excitation frequencies. However, the phase contrast itself reaches high values where the sample shows significant absorption due to the LPPhP excitation. Thus, we are unable to calculate the dispersion but the phase contrast spectrum is still retrievable. Such a phase spectrum corresponds to the excitation of a polariton available at a specific frequency. This spectrum can be reproduced by integrating all the momentum components at each excitation frequency by integrating the phase values along the horizontal lines of the calculated dispersion map. The momentum components in the calculation have to be weighted to match the measurements. For this, we applied a Gaussian window (details in Supporting Information section 7). In Figure 4 we plot the theoretical phase spectrum in blue along with two experimentally obtained phase spectra (green and red). One of the measured spectra (green) was calculated the same way as described above but from the experimental dispersion map. The other spectrum (red) was measured by calculating the average phase contrast along the nanotube. The result qualitatively matches the experimentally obtained spectrum; however, the small difference at low frequencies suggests that a silica–silicon interface phonon mode also has an effect on the LPPhP spectrum.

**Figure 4:**
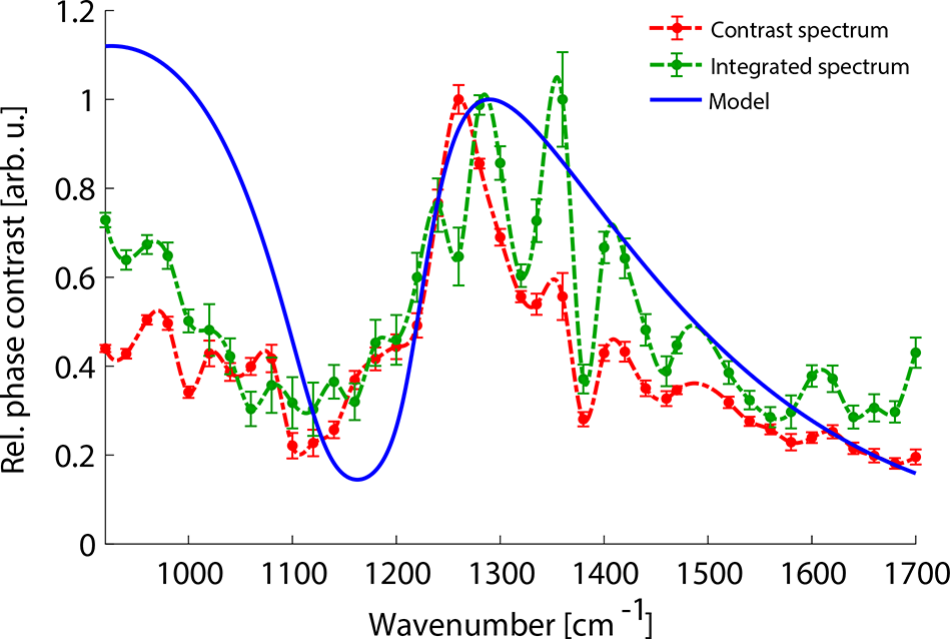
Excitation spectrum of Luttinger-liquid plasmon–phonon polaritons acquired from interference fringes by either taking the average phase contrast (red) or calculating from the dispersion map integrating all the Fourier components for each excitation frequency (green). The solid blue line is calculated from the theoretical dispersion map. Both spectra were normalized to their maximum between 1200 cm^–1^  and 1300 cm^–1^  to fit on a common scale. Red and green dashed lines are only guides to the eye.

We also recorded the phase images of the nanotube on the hBN flake (bottom parts of Figure 1d). Except for a few cases, the polariton interference fringes are not observable properly to reveal the dispersion; however, the spectral variation of the near-field phase contrast is clearly detectable. We plot the phase spectrum in Figure 5b.

**Figure 5:**
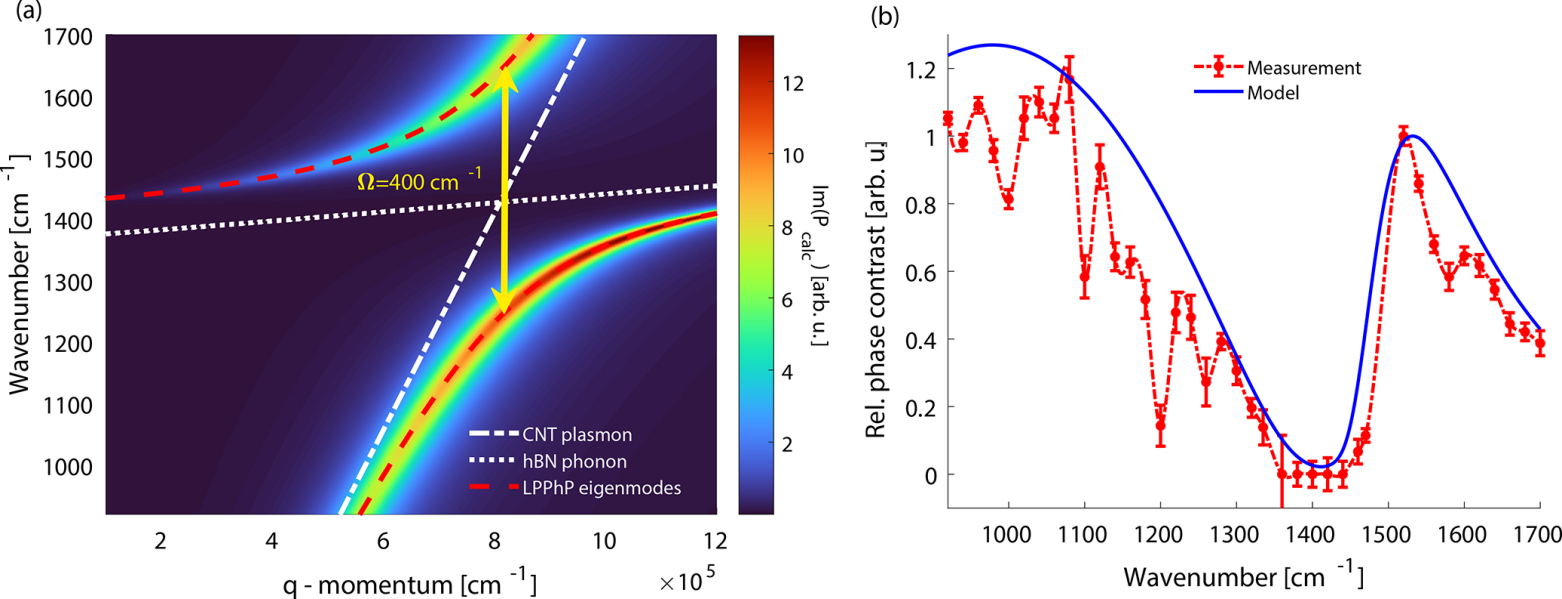
(a) Dispersion map of a LPPhP formed by the interaction of an hBN phonon and a nanotube Luttinger-liquid plasmon calculated via the coupled harmonic oscillator model. To correctly reproduce the experimental spectrum (b), the coupling strength had to be enhanced to *g* = 200 cm^–1^ which shows an even stronger coupling with the hBN phonons. The white dotted line shows the dispersion of the hBN slab phonon mode acquired from Im(*r*) (calculated via transfer matrix method). The white solid line represents the dispersion of the bare Luttinger-liquid plasmon. The red dashed lines present the eigenmode frequencies of the LPPhPs hybrid polariton given by eq 3. (b) Relative phase contrast spectrum of nanotube-hBN plasmon–phonon polaritons representing their excitation spectrum. Red dots are the experimental phase contrast values, and the solid blue line depicts the theoretical spectrum obtained from (a). Both spectra were normalized to their maximum above 1400 cm^–1^  to plot on a common scale. The red dashed line is only a guide to the eye.

We observe that the position of the spectral dip shifted to around 1400 cm^–1^. The absence of the spectral dip at around 1150 cm^–1^ proves that the 6 nm thick hBN slab separates the nanotube from the silica sufficiently that its phonon mode cannot interact with the nanotube plasmon; instead, the hBN phonon forms the hybridized states. To reproduce the phase spectrum theoretically, we calculated the Fresnel reflection coefficient of the 6 nm thick hBN slab via the transfer matrix method (see Supporting Information section 8). From the maxima of Im(*r*_hBN_) we retrieved the frequency of the slab mode. With the phonon oscillator values, we applied the coupled harmonic oscillator model (the theoretical dispersion map is shown in Figure 5a) and calculated the excitation spectrum of the new hybridized state. We found an increased coupling strength *g* = 200 cm^–1^  to fit the experimental spectrum. With the higher value of *g* and lower value of phonon damping γ_hBN_ = 5 cm^–1^ and mid-gap frequency 1427 cm^–1^, the value for the strong coupling criterion becomes *C* = 14.2 and the normalized coupling strength is η = 0.14. The calculated spectrum is shown in blue in Figure 5b and is in good agreement with the measurement.

In conclusion, our study demonstrates the unique properties of Luttinger-liquid plasmons in individual metallic carbon nanotubes to realize strong coupling in the mid-infrared regime. Due to their high concentration of electromagnetic fields, propagating Luttinger-liquid plasmons couple very effectively to thin layer phonon modes. We observed the transparency gap, opened by the hybridization, in real space by the disappearing near-field phase contrast of the nanotubes on top of as thin as 2 nm native silica. We used near-field polariton interferometry to reveal the dispersion of the hybrid plasmon–phonon mode, analyzed the coupling strength by the classical harmonic oscillator model, and determined that the normalized coupling strength reaches the ultrastrong coupling regime. A separation of the nanotube from the silica surface by 6 nm hBN completely removes the effect. Instead of silica, hBN phonons participate in the coupling, yielding an even stronger effect. Carbon nanotubes are promising candidates as building blocks for photonic nanocircuitry,^62^ and our study showed that a phononic substrate could add further customizability to the properties of nanotube-based circuits. Near-field polariton interferometry could allow tracking reactions of nanotube encapsulated molecules by vibrational strong coupling to Luttinger-liquid plasmons and thus open the way to ultrasensitive vibrational nanoanalytics.

## Supporting Information Available

Comparison of near-field amplitude and phase maps, PsHet setup schematics, sample preparation details, Lorentz oscillator parameters for hBN and silica, and details on the fitting procedure for nanotube plasmon damping, classical coupled oscillator model, spectrum calculation method, and phase shift upon polariton reflection (PDF)
