# Supplementary material for: Direct Visualization of Ultrastrong Coupling between Luttinger-Liquid Plasmons and Phonon Polaritons
Source: Nano Lett. 2022 Mar 22;22(8):3495–502. doi: 10.1021/acs.nanolett.1c04807 (PMC9052744; doi:10.1021/acs.nanolett.1c04807)
Supplement: Supplementary file 1 — nl1c04807_si_001.pdf [file nl1c04807_si_001.pdf]

Supplementary information for:

Direct visualization of ultrastrong coupling between Luttinger-liquid plasmons and phonon polaritons

Gergely Németh[1,2], Keigo Otsuka[3], Dániel Datz[1,4], Áron Pekker[1], Shigeo Maruyama[3], Ferenc Borondics[5], and Katalin Kamarás[1]

[1] *Wigner Research Centre for Physics, Konkoly Thege Miklós út 29-33., 1121 Budapest, Hungary*

[2] *Budapest University of Technology and Economics, Műegyetem rkp. 3., 1111 Budapest, Hungary*

[3] *Department of Mechanical Engineering, The University of Tokyo, Tokyo 113-8656, Japan*

[4] *Eötvös Loránd University, Pázmány Péter sétány 1/A, 1117 Budapest, Hungary*

[5] *Synchrotron SOLEIL, L'Orme des Merisiers, 91192 Gif Sur Yvette CEDEX, France*

1 Near-field amplitude and phase contrast of individual carbon nanotubes

In our study we only used near-field phase maps for the data processing. The near-field amplitude values measured at a specific pixel position depend sensitively on the tip-substrate separation. As the tip-sample separation increases, the amplitude value drops. Small non-resonant residues on the surface cause lower local amplitude values. This can alter the amplitude along the nanotube as well, yielding false, misleading artifacts. Fig. S1 presents the (a) AFM topography, (b) 4th harmonic amplitude map, (c) 4th harmonic phase map of the nanotube of interest after two days of measurements. Small dark spots in the amplitude map are well recognizable in the topography. On the hexagonal boron nitride (hBN) flake (bottom part), we can also see a big blob of dirt collected by the tip causing a serious artifact in the amplitude map but having only a small effect on the phase measurements.

As mentioned in the main text, the contrast of the nanotube was normalized to the values of the underlying substrate both for the silica and hBN region. In order to make this contrast correction easier during data processing, the fast scan direction (horizontal axis) was set parallel to the material boundary.

The post-processing consisted of the following steps (in the case of amplitude correction a similar process can be used, replacing the subtraction operation by division):

1. Align rows: calculating a representative value (here median was used) of each line and subtracting it from all pixel values in the line. It is a very common scan line artifact correction method in SPM techniques. Because the sample is oriented in a way that the material boundary is horizontal, the median value of a line is representing the specific substrate level.

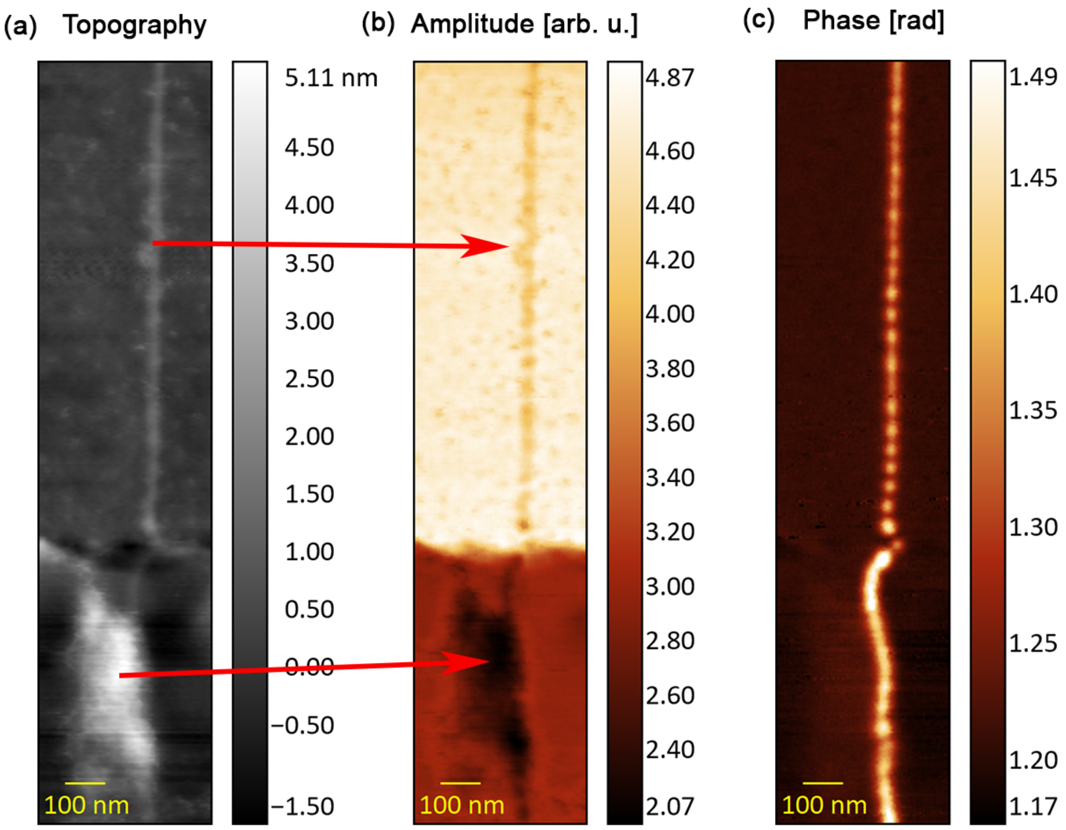

Figure S1: (a) AFM topography (b) Near-field amplitude (c) Near-field phase map of the area of interest. The optical images were taken at 920 cm^{-1}

2. Plane leveling: the normalization then happened with a plane subtraction. It removes any remaining tilt and shifts the base value to 0.

3. The phase profiles were extracted along the nanotube starting from the most intense maximum of the interference pattern as demonstrated in Fig. 1 (c) in the main text.

4. The Fourier spectrum of the line profile was calculated by FFT giving the spatial frequency (f) of the oscillation pattern. The plasmon wavelength can be calculated by $\lambda_p = 4\pi/f$.

5. The plasmon wavelength was acquired also by measuring the distances between each adjacent maxima through the whole line profile. The average and the standard deviation of these values were calculated.

2 Details on sample preparation

Our samples consist of horizontally aligned single-walled carbon nanotubes (SWCNTs) on silicon substrate with hBN flakes. The silicon wafer has a 2 nm native oxide layer that is naturally present on the surface. The samples were prepared in the following way:

1. hBN exfoliation: hBN flakes were mechanically exfoliated from a large piece of hBN crystal

(HQgraphene, h-BN, BN2A1) using blue nitto tape (Nitto Denko Co., SPV 224P). The tape with the exfoliated flakes was brought in contact with the silicon subtrate, then peeled off leaving a number of hBN flakes with a variety of size and thickness.

2. CNT growth: SWCNTs were grown by chemical vapor deposition technique on an r-cut quartz substrate using iron catalyst nanoparticles described in detail in [1].

3. CNT transfer: 4 wt% PMMA solution in anisole was spin-coated at 2000 rpm on the SWNTs. The substrates were heated at 170 °C in air for 15 min and then immersed in heated aqueous KOH (1 mol/L, 100 °C). The PMMA film was peeled off from the substrate in cold distilled water and picked up by the substrates. The substrate was annealed at 170 °C for 15 min. The PMMA layer was dissolved using acetone followed by a 3 hours long annealing in Ar/H_2 atmosphere at 350 °C.

The position of the nanotubes and the hBN flakes are random. The appropriate nanotube was selected based on large area SNOM scans.

3 Pseudo-heterodyne s-SNOM setup

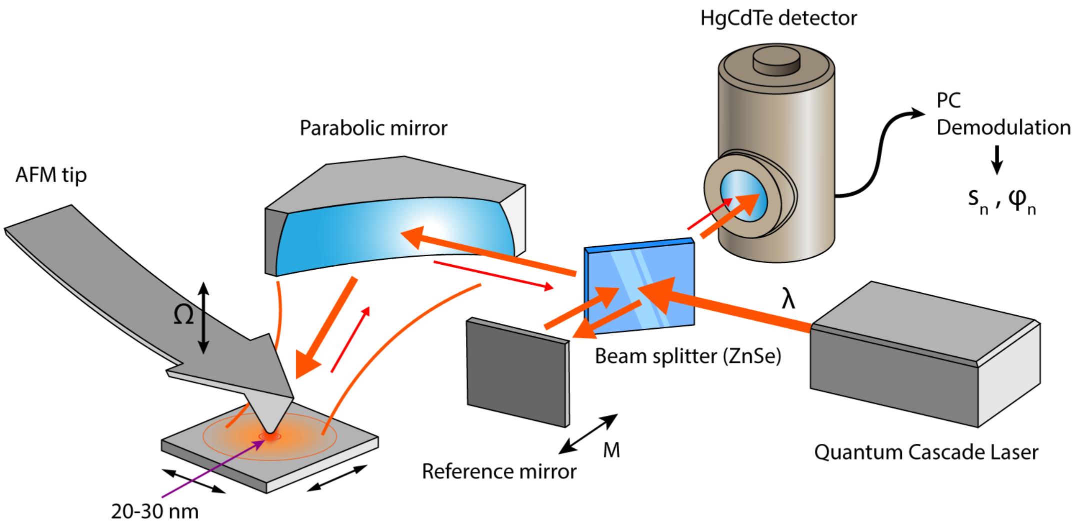

Figure S2: Schematic illustration of the pseudo-heterodyne s-SNOM setup.

Our instrument (NeaSNOM by NeaSpec GmbH) applies the so-called pseudo-heterodyne (PsHet) detection scheme. The illustration of such a setup can be seen in Fig. S2. This detection method allows to separate the pure near-field signal from the background scattering and additionally makes it possible to measure the amplitude and the phase of the scattered waves originating from the near-field interaction. The setup consists of the AFM part with focusing optics and an asymmetric Michelson interferometer as depicted below. The detailed derivation and description of this method can be found in the following references that created the basis to realize such an instrument: [2, 3, 4].

4 Dielectric function of silica and hexagonal boron nitride

In our study we used a Lorentz dielectric function model to describe the optical response of silica given by:

$$\epsilon(\omega) = \epsilon_\infty + \sum_i \frac{\omega_{pi}^2}{\omega_i^2 - \omega^2 - i\gamma_i\omega} \tag{1}$$

In the spectral region of interest we used only one oscillator, with $\epsilon_\infty = 1.88$, $\omega_1 = 1071.9$ cm^{-1}, $\gamma_1 = 29.9$ cm^{-1}. The parameters were found by fitting the dielectric function values in the SI of [5].

In case of hBN, since it is highly anisotropic, its dielectric function is different for the in-plane and out-of-plane direction:

$$\epsilon_a(\omega) = \epsilon_{a,\infty}\left(1 + \frac{\omega_{a,LO}^2 - \omega_{a,TO}^2}{\omega_{a,TO}^2 - \omega^2 - i\gamma_a\omega}\right) \tag{2}$$

where $a = \perp, \parallel$ and $\epsilon_{\perp,\parallel,\infty} = 4.52$, $\omega_{\parallel,TO} = 746$ cm^{-1}, $\omega_{\parallel,LO} = 819$ cm^{-1} $\omega_{\perp,TO} = 1360$ cm^{-1}, $\omega_{\perp,LO} = 1610$ cm^{-1}, $\gamma_\parallel = 4$ cm^{-1}, $\gamma_\perp = 5$ cm^{-1}. The values were taken from [6].

5 Fitting nanotube oscillator parameters

In order to apply the harmonic oscillator model, we have to acquire the parameters of each oscillator participating in the coupling. For the silica layer the parameters are straightforward to obtain as described in the main text. For the nanotube, it is more difficult because we do not know the dielectric tensor. However, we know the linear dispersion relation from which we calculate an oscillator's eigenfrequency. For the damping γ_{cnt} we have to rely on the experimental dispersion map. To obtain the parameters, one could fit a vertical line cut from the dispersion map and fit the experimental data with the imaginary part of a Lorentz oscillator $y = A^2/(\omega_i^2 - \omega^2 - i\gamma\omega)$. As the experimental data suffer from large uncertainty, we used a vertical quasi-line cut. We take a narrow area as shown by the red square in Fig. S2 (a). By averaging the values in the q direction, we can enhance the signal-to-noise ratio a bit. The resulting quasi-line cut and the corresponding fit can be seen in Fig. S2 (b). The acquired damping value is $\gamma_{cnt} = 150$ cm^{-1}. We note that this method overestimates the damping as the averaging broadens the experimental curve. This effect is, however, not really significant. By repeating the same procedure for a theoretical dispersion map we estimate the broadening and overestimation of γ_{cnt} by only around 10% which does not alter our findings about the regime of coupling significantly.

Since one could argue that the oscillator parameter could not be the bare one because we are not far enough from zero detuning, we also calculated the nanotube plasmon oscillator damping in a different way.

We took a few vertical lines around zero detuning as shown in Fig. S3a by the red rectangular area. The resulting function of polariton amplitude versus frequency can be sen in Fig. S3b. One can clearly observe the two hybrid polariton modes with the splitting consistent with the values in the main text. We fitted this spectrum with the sum of two Lorentzian functions described above. This yields a very good fit as plotted in Fig. S3 (b) by the red curve. In case of zero detuning, the damping of the hybrid polariton is $\gamma_{pol} = (\gamma_{cnt} + \gamma_{SiO2})/2$. Utilizing this formula we can also

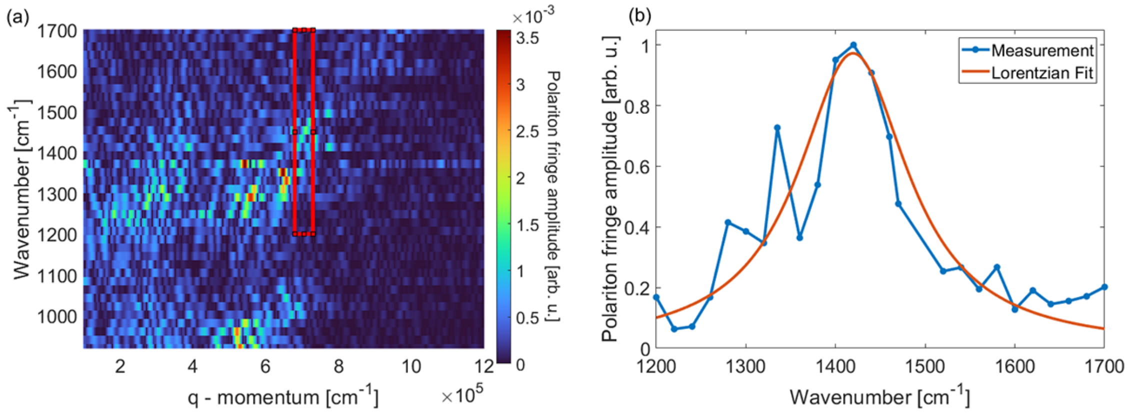

Figure S3: (a) Experimental dispersion of Luttinger-liquid plasmon-phonon polaritons. Each row represents the Fourier spectrum of the polariton fringe profile taken at the corresponding frequency. The red box marks the area that was cut out and used to fit the damping of the nanotube oscillator. (b) Lorentzian fit to the average frequency curve obtained from the area marked by the red box.

calculate the nanotube plasmon damping because the SiO_2 phonon damping is known. The fit provided $\gamma_{pol} = 90$ cm^{-1} and using $\gamma_{SiO2} = 30$ cm^{-1}, we get the same value for the nanotube damping $\gamma_{cnt} = 150$ cm^{-1}. The acquired splitting from the position of the fitted Lorentzians is also consistent with the $\Omega = 300$ cm^{-1} value from the dispersion fitting.

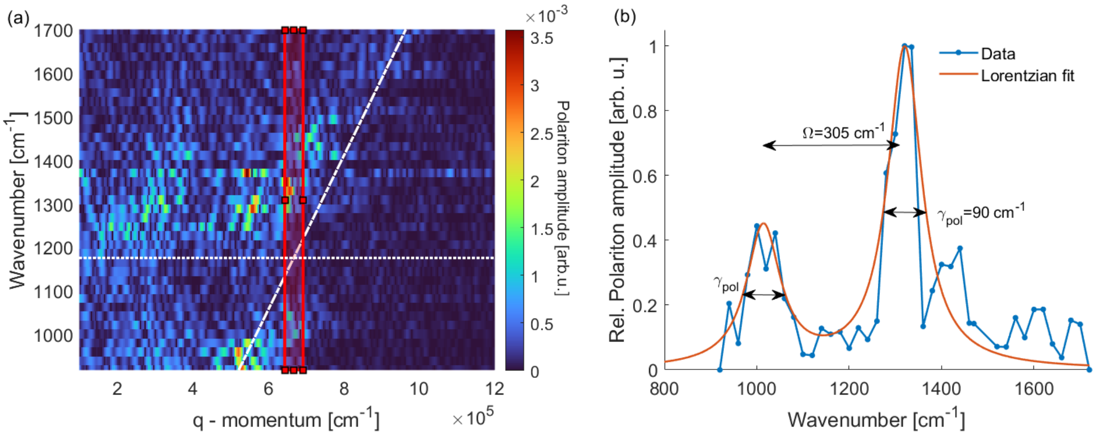

Figure S4: (a) Experimental dispersion of Luttinger-liquid plasmon-phonon polaritons. Each row represents the Fourier spectrum of the polariton fringe profile taken at the corresponding frequency. The red box marks the area that was cut out and used to fit the damping of the hybrid polariton modes. (b) Blue points show the average polariton amplitude versus frequency curve obtained from the area marked by the red box. The fitted curve consisting of two Lorentzian functions is plotted in red.

6 Classical coupled oscillator model

We describe the coupling of nanotube plasmons and phonon polaritons via a classical model of coupled oscillators. For sake of simplicity we assume that only the nanotube plasmon is driven. The equation of motion for each oscillator describing their coupling:

$$\ddot{x}_{cnt} + \gamma_{cnt}\dot{x}_{cnt} + \omega_{cnt}^2 x_{cnt} + 2g\dot{x}_{SiO_2} = \frac{e}{m}E_{loc} \tag{3}$$

$$\ddot{x}_{SiO_2} + \gamma_{SiO_2}\dot{x}_{SiO_2} + \omega_{SiO_2}^2 x_{SiO_2} + 2g\dot{x}_{cnt} = 0 \tag{4}$$

We seek the plane wave solution in the form $x = Ae^{-i\omega t}$, the equations become

$$\Gamma_{cnt}x_{cnt} + Kx_{SiO_2} = \frac{e}{m}E_{loc} \tag{5}$$

$$\Gamma_{SiO_2}x_{SiO_2} - Kx_{cnt} = 0 \tag{6}$$

where $\Gamma_j = \omega_j^2 - i\omega_j - \omega^2$, j stands for either nanotube (cnt) or silica (SiO_2) and $K = 2ig\omega$. Solving for the steady state solution of the equation system, for x_{cnt} the result becomes

$$x_{cnt} = \frac{f \cdot \Gamma_{SiO_2}}{\Gamma_{cnt}\Gamma_{SiO_2} + K^2} \tag{7}$$

where $f \propto e/mE_{loc}$. From here the polarization induced in the nanotube is $P = e \cdot x$. We then visualize the dispersion map as Im(P). As can be seen in Fig. 3. it truly describes the experimental map and the spectra calculated using this theoretical map as described in Section S7.

7 Spectrum calculation from the dispersion map

When polariton interferometry cannot be applied to measure experimental dispersion, the coupling can be extracted from the phase contrast spectrum of the nanotubes. At a specific illumination frequency, the tip launches every plasmon when its momentum is matching the available momentum range of the tip's near field. The phase contrast measured at a given illumination frequency contains the contributions from all these plasmon excitations. A quantity that is proportional to the measured phase contrast can be replicated by summing all $\mathrm{Im}(P_{calc}(q,\omega))$ values calculated at a given frequency by taking into account the weights of the momentum components. We found that a Gaussian weight function $W_q(\omega)$ applied to each row of the dispersion map replicates the experimental phase spectrum quite well (main text Fig. 4 and Fig. 5b). We set the position of the Gaussian weight function to $q = 5.5 \cdot 10^5$ cm^{-1}, as this choice matches the experimental dispersion and replicates the contrast spectrum well. The Gaussian function applied to the theoretical dispersion map is shown in Fig. S5. The theoretical phase contrast spectrum $C_\varphi(\omega)$ thus calculated as

$$C_\varphi(\omega) = \int_0^\infty W(q) \cdot P_{calc}(q,\omega)\, dq \tag{8}$$

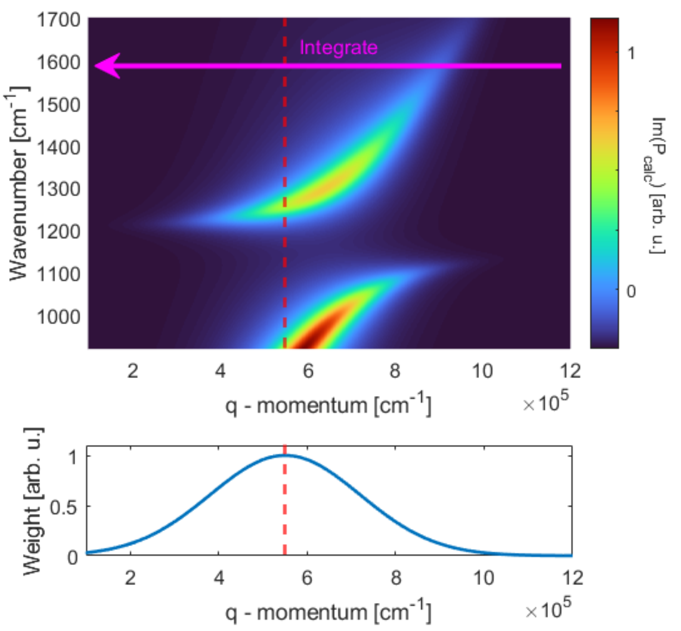

Figure S5: Theoretical dispersion map based on the classical harmonic oscillator model. A Gaussian weight function was applied for each row. The position of the weight function is marked by the dashed red line. The purple arrow represents that the spectrum is calculated by integrating the contribution of each q component.

8 Fresnel reflection coefficient of a 6 nm thick hBN flake

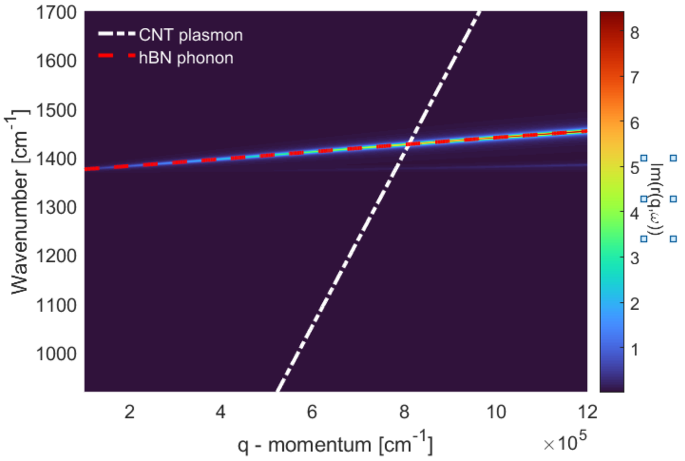

Figure S6: Imaginary part of the Fresnel reflection coefficient of a 6 nm thick hBN flake on top of Si. The red dashed line shows the frequencies acquired from the maxima of the main slab mode. The white dashed line represents the dispersion of the bare Luttinger liquid plasmon. In the case of the carbon nanotube lying on top of such an hBN slab, these are the interacting oscillators.

9 Reflection phase shift of nanotube plasmons

In our measurements the polariton fringes are created by the forward propagating and reflected polaritons. Upon polariton reflection a phase shift can occur on the reflected wave. The reflection phase shift (RPS) causes variation in the interference fringe profile mostly in the proximity of the reflecting artifact. In our case its origin can be rather complex since there is a geometric discontinuity (arbitrary change in the shape of the nanotube as it runs up to the hBN flake) and a change in dielectric environment. In the near-field phase maps one can notice a reflection phase shift (RPS), similarly to previous studies [7]. The first maximum of the interference fringe has lower intensity than the second one (see Fig. S1 (c)). This is a mark of reflection phase shift. Our analysis did not focus on RPS because the geometry here is not ideal to a precise study, however, we measured the value of RPS at two distinct frequencies (920 cm^{-1}, 1400 cm^{-1}). We fit the phase profiles with a damped oscillator form of $A \cdot e^{-2\pi x/(Q\lambda_p} \cos{(4\pi x/\lambda_p + \Phi)}$, where x is the distance from the nanotube end, Q is the quality factor, λ_p is the polariton wavelength, Φ is the reflection phase shift. The fit in both cases is plotted in Fig. S7. The acquired values of $\mathrm{RPS}_{920} = 0.08\pi$ and $\mathrm{RPS}_{1400} = 0.38\pi$, which correlates with the previously reported values in the range of the accuracy of the reported study.

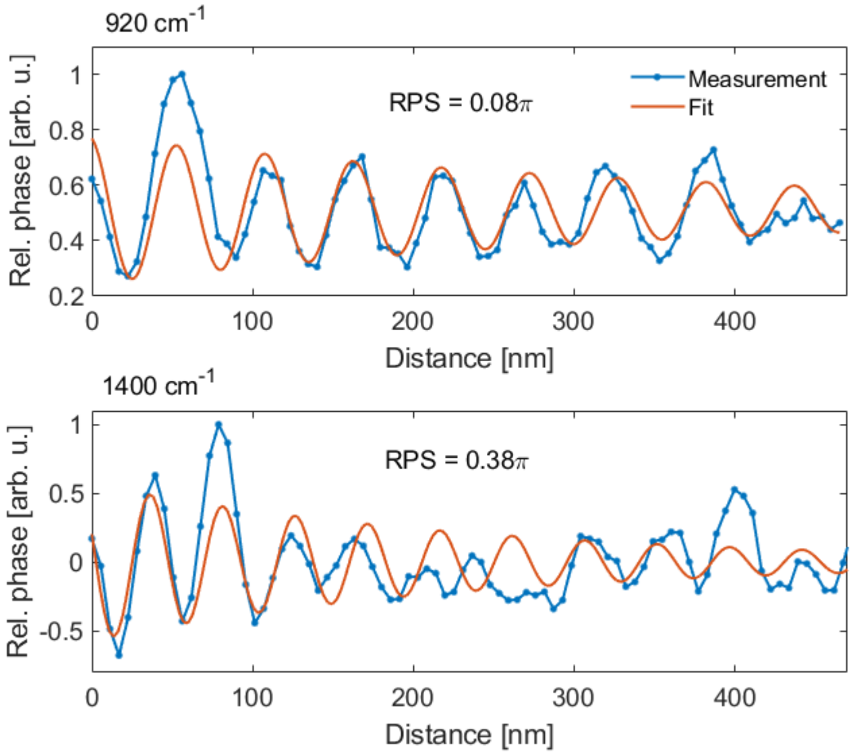

Figure S7: Reflection phase shift of the polariton waves from the nanotube end measured at two different wavenumbers. The fitting to a damped oscillator with an additional phase term results in $\mathrm{RPS}_{920} = 0.08\pi$ and $\mathrm{RPS}_{1400} = 0.38\pi$ which is in agreement with reported values in the literature within the range of accuracy [7].

10 Reliability of the measurements

As it is shown in the main text the widely accepted criterion used for strong coupling is written as $C \equiv \Omega^2/\left(\frac{\gamma^2_{\rm CNT}}{2} + \frac{\gamma^2_{SiO2}}{2}\right) > 1$, where $\Omega = 2g$ (mode splitting)[8, 9, 10]. The obtained values of $g_{SiO2} = 150$ cm^{-1} leads to $C_{SiO2} = 7.7$, far beyond the limit of $C > 1$.

With the uncertainty of the obtained value of g, one could argue that, the criterion for strong coupling might not be satisfied at some point. Let us examine now the lower limit of strong coupling based on the criterion for C. We can say that the transition between weak and strong coupling is $C = 1$. From this statement, it follows for the value of g:

$$g = \frac{1}{2}\sqrt{\frac{\gamma^2_{CNT}}{2} + \frac{\gamma^2_{Ph}}{2}}, \tag{9}$$

where γ_{Ph} is the damping of the specific phonon mode for silica or hBN. This worst case scenario will lead to $g_{SiO2} = 54.1$ cm^{-1}. With these values we can replot the dispersion curves and the polariton spectrum to compare with the data and to visualize how far the strong coupling limit is from reality. The yellow dashed line in Fig. 10.1 (a) shows the dispersion curve for a system with the above provided coupling strength. As can be clearly seen from the figure, it does not matter if we consider the data low or high quality, these low g curves deviate considerably from the measurement. Furthermore, we also presented the splitting at zero-detuning both in the main text and in the supplementary information (Section S5). Here again, the yellow solid line in Fig. 10.1 (b) presents how the zero-detuning spectrum would look like in the case of $g_{SiO2} = 54.1$. It is obvious that g must have higher values, thus the coupling strength is unquestionably higher than it is required for the strong coupling ($C > 1$). We strongly believe that the presented explanation is scientifically sound, the data are clear, regardless of the noise and they prove our original statement unambiguously.

Regarding ultrastrong coupling (USC), it has stricter requirements and we will discuss this below.

In case of USC, the damping does not come into play at all, as it is defined by the normalized coupling strength $\eta = g/\omega_g \geq 0.1$ where ω_g is the midgap frequency[11]. Thus, the lower limit for USC is $\eta = 0.1$ which would translate to $g_{SiO2} = 117.6$ cm^{-1}. The orange lines in Fig. 10.1 (a) and (b) show the dispersion curve and the zero-detuning spectrum for these values of g. Already, the comparison of the experimental dispersion and the new low g curves point out that all the intense peaks of the data are above the curve which proves that the applied g is too low. If it is not convincing enough, the zero-detuning spectrum similarly, probably even more convincingly, shows that the splitting is higher than $\Omega = 2g = 235.2$ cm^{-1}. The automated fitting provided the red curve with splitting $\Omega = 305$ cm^{-1}, which we even rounded down to 300 cm^{-1}. Once again, the evidence is clear regardless of the relative data quality.

Here we comment on the measurements and results on the nanotube-hBN part. We show that the derived contrast spectrum can provide the value of g reliably enough to strongly suggest USC and undoubtedly SC. We repeat the approach presented in the previous section for silica but presenting the contrast spectrum. In case of the hBN phonon mode the USC criterion, $\eta = 0.1$, would mean $g = 142.7$ cm^{-1}, while the SC criterion $C = 1$ would mean $g = 53.1$ cm^{-1}. For various cases we calculated the contrast spectrum the same way as in the manuscript and plotted the results in Fig. 10.2. The conclusions can be easily observed. Even the required minimum value for USC results

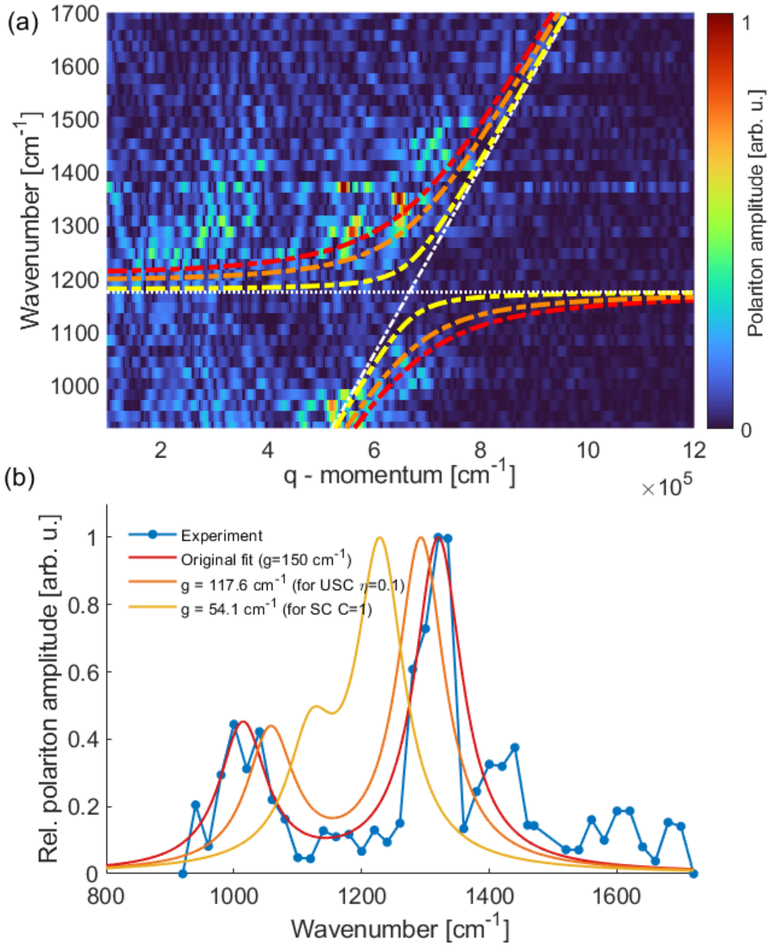

Figure S8: Dependence of the model fit on the coupling strength with several g values as determined by either η=0.1 USC regime (orange) or C=1 (yellow) SC regime. (a) Experimental dispersion map with the dispersion curves overlayed. (b) Zero-detuning experimental polariton spectrum (blue) and different scenarios with the crucial g values representing USC or SC regime. The red line represents the original fit to the data providing $\Omega = 305$ cm^{-1}.

in a spectrum significantly different from the measurements. Modeling only SC results in an even worse match between the model and experiment regardless of the signal-to-noise ratio.

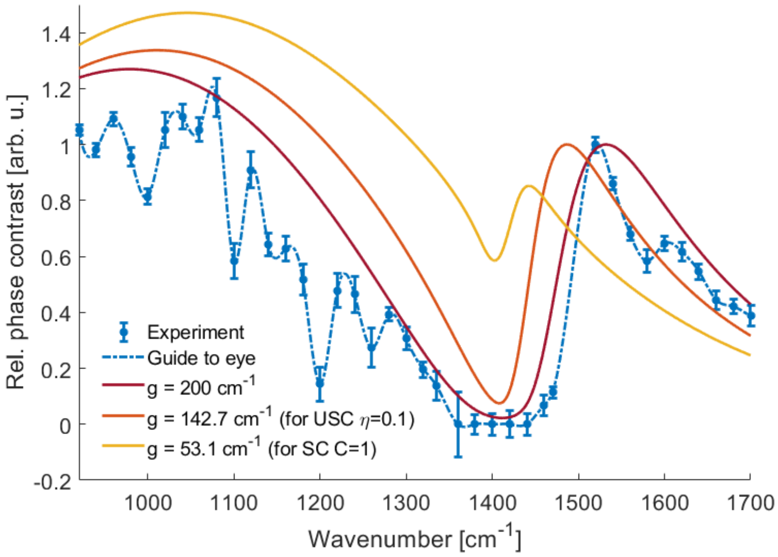

Figure S9: Contrast spectrum of the nanotube on the 6 nm hBN flake. The blue points are the measured phase contrast values. The red spectrum plots the original fit with $g = 200$ cm^{-1}. The orange and yellow spectrum shows the calculated spectrum in case of decreased $g = 142.7$ cm^{-1} presenting the situation of $\eta = 0.1$ while the yellow line shows the case if SC criterion $C = 1$ would play out with $g = 53.1$ cm^{-1}.

References

[1] K. Otsuka, T. Inoue, E. Maeda, R. Kometani, S. Chiashi, and S. Maruyama, "On-Chip sorting of long semiconducting carbon nanotubes for multiple transistors along an identical array," *ACS Nano*, vol. 11, pp. 11497–11504, Nov. 2017.

[2] N. Ocelic, A. Huber, and R. Hillenbrand, "Pseudoheterodyne detection for background-free near-field spectroscopy," *Appl. Phys. Lett.*, vol. 89, no. 10, p. 101124, 2006.

[3] N. Ocelic, *Quantitative Near-field Phonon-polariton Spectroscopy*. PhD thesis, Technical University Munich, 2007.

[4] M. Vaez-Iravani and R. Toledo-Crow, "Phase contrast and amplitude pseudoheterodyne interference near field scanning optical microscopy," *Appl. Phys. Lett.*, vol. 62, no. 10, pp. 1044–1046, 1993.

[5] L. M. Zhang, G. O. Andreev, Z. Fei, A. S. McLeod, G. Dominguez, M. Thiemens, A. H. Castro-Neto, D. N. Basov, and M. M. Fogler, "Near-field spectroscopy of silicon dioxide thin films," *Phys. Rev. B*, vol. 85, p. 075419, Feb. 2012.

[6] M. Autore, P. Li, I. Dolado, F. J. Alfaro-Mozaz, R. Esteban, A. Atxabal, F. Casanova, L. E. Hueso, P. Alonso-González, J. Aizpurua, A. Y. Nikitin, S. Vélez, and R. Hillenbrand, "Boron nitride nanoresonators for phonon-enhanced molecular vibrational spectroscopy at the strong coupling limit," *Light Sci. Appl.*, vol. 7, no. 4, pp. 17172–1–8, 2018.

[7] X. Luo, C. Hu, B. Lyu, L. Yang, X. Zhou, A. Deng, J.-H. Kang, and Z. Shi, "Reflection phase shift of one-dimensional plasmon polaritons in carbon nanotubes," *Phys. Rev. B*, vol. 101, p. 041407, Jan. 2020.

[8] P. Törmä and W. L. Barnes, "Strong coupling between surface plasmon polaritons and emitters: a review," *Rep. Prog. Phys.*, vol. 78, p. 013901, Jan. 2015.

[9] A. Bylinkin, M. Schnell, M. Autore, F. Calavalle, P. Li, J. Taboada-Gutièrrez, S. Liu, J. H. Edgar, F. Casanova, L. E. Hueso, P. Alonso-Gonzalez, A. Y. Nikitin, and R. Hillenbrand, "Real-space observation of vibrational strong coupling between propagating phonon polaritons and organic molecules," *Nat. Photonics*, vol. 15, pp. 197–202, Nov. 2020.

[10] M. Barra-Burillo, U. Muniain, S. Catalano, M. Autore, F. Casanova, L. E. Hueso, J. Aizpurua, R. Esteban, and R. Hillenbrand, "Microcavity phonon polaritons from the weak to the ultrastrong phonon–photon coupling regime," *Nat. Commun.*, vol. 12, p. 6206, Dec. 2021.

[11] A. Frisk Kockum, A. Miranowicz, S. De Liberato, S. Savasta, and F. Nori, "Ultrastrong coupling between light and matter," *Nat. Rev. Phys.*, vol. 1, pp. 19–40, Jan. 2019.
